# Fractal-like kinetics of intracellular enzymatic reactions: a chemical framework of endotoxin tolerance and a possible non-specific contribution of macromolecular crowding to cross-tolerance

**DOI:** 10.1186/1742-4682-10-55

**Published:** 2013-09-14

**Authors:** Catalin Vasilescu, Mircea Olteanu, Paul Flondor, George A Calin

**Affiliations:** 1 Fundeni Clinical Institute, Carol Davila University of Medicine and Pharmacy, 258 Fundeni Street, Bucharest, Romania; 2 Department of Mathematical Methods and Models, Politehnica University, Bucharest, Romania; 3 Department of Experimental Therapeutics, Unit 1950, The University of Texas MD Anderson Cancer Center, 1515 Holcombe Boulevard, Houston, TX 77030, USA

**Keywords:** Endotoxin tolerance, Fractal-like kinetics, Macromolecular crowding, Mathematical models, Power law, Sepsis

## Abstract

**Background:**

The response to endotoxin (LPS), and subsequent signal transduction lead to the production of cytokines such as tumor necrosis factor-α (TNF-α) by innate immune cells. Cells or organisms pretreated with endotoxin enter into a transient state of hyporesponsiveness, referred to as endotoxin tolerance (ET) which represents a particular case of negative preconditioning. Despite recent progress in understanding the molecular basis of ET, there is no consensus yet on the primary mechanism responsible for ET and for the more complex cases of cross tolerance. In this study, we examined the consequences of the macromolecular crowding (MMC) and of fractal-like kinetics (FLK) of intracellular enzymatic reactions on the LPS signaling machinery. We hypothesized that this particular type of enzyme kinetics may explain the development of ET phenomenon.

**Method:**

Our aim in the present study was to characterize the chemical kinetics framework in ET and determine whether fractal-like kinetics explains, at least in part, ET. We developed an ordinary differential equations (ODE) mathematical model that took into account the links between the MMC and the LPS signaling machinery leading to ET. We proposed that the intracellular fractal environment (MMC) contributes to ET and developed two mathematical models of enzyme kinetics: one based on Kopelman’s *fractal*-*like kinetics* framework and the other based on Savageau’s *power law* model.

**Results:**

Kopelman’s model provides a good image of the potential influence of a fractal intracellular environment (MMC) on ET. The Savageau power law model also partially explains ET. The computer simulations supported the hypothesis that MMC and FLK may play a role in ET.

**Conclusion:**

The model highlights the links between the organization of the intracellular environment, MMC and the LPS signaling machinery leading to ET. Our FLK-based model does not minimize the role of the numerous negative regulatory factors. It simply draws attention to the fact that macromolecular crowding can contribute significantly to the induction of ET by imposing geometric constrains and a particular chemical kinetic for the intracellular reactions.

## Introduction

Endotoxin tolerance (ET) is a phenomenon in which cells or organisms exposed to an endotoxin (e.g., lipopolysaccharide [LPS]) at a low concentration enter a transient unresponsive state and are unable to respond to further challenges with endotoxin [1]. Researchers have observed this phenomenon both *in vitro* and *in vivo* in animal models as well as humans. ET is regulated at multiple levels, both transcriptionally and post transcriptionally, by genetic and epigenetic mechanisms such as methylation and noncoding RNA regulation [2]. However, the molecular basis for ET remains unclear. In many aspects, ET resembles immunodepression, immunosuppression, and immunoparalysis as reported in patients with sepsis or noninfectious systemic inflammatory response syndrome such as trauma, surgery, hemorrhagic shock [3], acute pancreatitis [4,5] and acute respiratory distress syndrome [6]. Tumor necrosis factor (TNF) is likely the best marker for ET as assessed because of its absence following LPS challenge in endotoxin-tolerized animals in contrast with the sharp, rapid peak in response of an initial injection of LPS [3,7]. Most investigators have tried to understand these phenomena by examining the action of different inhibitors that are low on the LPS signaling main reaction chain. Researchers have observed some important differences between *in vitro* and *in vivo* models of ET and sepsis [8]. In the present study, we sought to analyze these discrepancies and show that they at least partly result from particularities of the chemical kinetics of enzymatic reactions *in vivo*.

LPS signaling (cytokine release induced by LPS stimulation) can be considered a chain of enzymatic reactions. To find out something about ET, a close examination of the enzymatic reaction mechanisms and rates may be useful. Chemical kinetics entails the measurement of concentrations of reactants as a function of time with the goal of understanding and characterizing the enzymatic reaction mechanism [9,10]. However, much of the current cellular biochemistry paradigm is extrapolated from studies assuming ideal reaction conditions *in vitro*: an infinite reaction volume and dilute and homogenous solutions containing single enzymes and substrates [11]. Despite the physiological feature**s** of intracellular environment, biochemists commonly study the properties of macromolecules in these solutions with a total macromolecular concentration in which crowding is negligible [12]. In fact, most *in vivo* enzymatic reactions take place inside the cell, which is not an ideal reaction environment. The main characteristics of the intracellular environment that are not present *in vitro* biochemical reactions are macromolecular crowding and hindered diffusion by compartmentalization (heterogeneity) [11-13]. Diffusion of proteins *in vivo* is significantly lower than under dilute conditions.

The *in vivo* kinetic rate constants and even the structure of kinetic rate expression can differ significantly from those *in vitro* tests [14]. For example, in the cytoplasm of eukaryotic cells, diffusion of both large and small molecules is slowed down three to four times [15]. Bimolecular reactions are governed by molecule collisions. In turn, the frequency of these collisions depends on molecular mobility. Molecular crowding and, especially, the cytoskeleton structure lead to a reduction in the diffusion rate, which depends on the molecule size. Collision of molecules in diffusion-limited reactions translates into reduced enzymatic reaction rates [14,16,17].

Anomalous molecular diffusion, which occurs in crowded systems, leads to time-dependent reaction rate coefficients [14]. Simulating a crowded intracellular environment seems to be crucial to understanding the nature of living systems. Complications arise owing to cellular heterogeneity [15]. Authors have described other effects of this type of macromolecular crowding on molecule diffusion (e.g., hydrodynamic interactions, electrostatic forces) [18]. The excluded volume effect is probably the most important of them [12].

An emerging view of living cell cytoplasm is that it has a structured, organized macromolecular assembly. This complex architecture has profound consequences for cell function. A realistic picture of the cytoplasm looks very much like a network composed of actin filaments, microtubules, intermediate filaments, and associated proteins. A fractal view of the living cell cytoplasm is similar to the “structured” view but also includes new, likely behavioral possibilities [19]. Aon and Cortassa [19] and Forgacs et al. [20] suggested that the cytoplasm is organized as a percolation cluster, or a type of random fractal. This hypothesis is based on imaging and quantification of the fractal dimension of macromolecular associations *in vitro* and on published micrographs describing the cytoskeleton in cells.

Investigators also have defined anomalous diffusion by using a random walker on percolation clusters. The percolation theory deals with the number and properties of clusters. Each position in a very large lattice-type structure is occupied randomly by a molecule at probability *p* independent of the neighbors [21,22]. When *p* is higher than the critical value *p*_*c*_, the cluster reaches from one side of the lattice to the other. Anomalous diffusion in an inhomogeneous environment is observed when the reaction space is increasingly occupied heterogeneously by obstacles until the relative volume of obstacles nears the threshold [21,22]. In a living cell, the percolation lattice is determined by the cytoskeletal organization. If *p* is close to *p*_*c*_, this may be a fine-tuning mechanism of intracellular reaction velocity in that it modifies the enzyme kinetics of intracellular reactions via changes in the fractal dimension of the cytoplasm.

These particularities (specifically geometric constraints) are now accepted as having important implications regarding the kinetics of intracellular enzyme reactions. In two seminal studies Kopelman showed the classical reaction kinetics to be unsatisfactory when the reactants were spatially constrained at the microscopic level by either walls or phase boundaries [23,24].

Enzymatic reactions have exhibited well-studied chemical kinetics (Michaelis-Menten model of enzyme kinetics) *in vitro*. In a two-reactant bimolecular reaction (*A* + *B* product) with a dilute and homogenous solution (*in vitro* conditions), the reaction rate is *k* [A] [B], in which *k* is a constant and [A] and [B] are concentrations. Investigators have used two mathematical approaches describing this particular type of reaction kinetics. 1) According to Kopelman [24], for enzymatic reactions in nonhomogeneous (fractal) environments, the reaction rate coefficients are not time-independent and are given by $kt^{-h},0\;\leq \;h\;\leq \;1,\;t\;\geq \;1$, with a constant *k*. Thus, in diffusion-limited reactions, the reaction rate is time-dependent. Kopelman called this fractal-like kinetics. 2) In comparison, according to Savageau [25], approximately equivalent results can be obtained by using so-called power law kinetics, which is discussed below.

The consequences of fractal-like kinetics (owing to macromolecular crowding) on the reaction rate are twofold. 1) In the beginning of the reaction, the reaction rate is higher than expected according to the classical Michaelis-Menten model (for a short period, the reaction is more efficient). 2) Afterward, the reaction rate is slower than if it were taking place in a homogenous environment.

The term fractal environment may create some confusion. We suggest that the term anomalous diffusion, which is well accepted in the field of physics, is a more accurate description of the intracellular enzymatic reaction conditions (or perhaps geometrical constraints for *in vivo* reactions). However, we are interested in enzymatic reactions *in vivo*, but the same model can be used for all chemical reactions if researchers agree that the reactions result from collision of reactant molecules [16].

In the present study, we examined the consequences of fractal-like kinetics of intracellular enzymatic reactions for the LPS signaling machinery. To do so, we tested our hypothesis that this particular type of enzyme kinetics may explain ET and immunosuppression in septic patients. We discuss ET in light of these new findings and attempt to take an integrated view of ET.

### Mathematical models, simulations and results

#### Preliminaries

In previous studies, researchers examined ET in a simple model based on the Michaelis-Menten-Hill model [4,8,26]. In the present study, we built mathematical models of ET based on fractal-like kinetics according to Kopelman’s model of fractal-like kinetics and Savageau’s power law model. First, in Kopelman’s version of fractal-like kinetics, the rate coefficient is expressed as *k*_1_(*t*) = *k*_1_*t*^− *h*^[23]. Our approach considers elimination of the product of the reaction. In our version of Savageau’s model [25,27], time-dependent exponents are considered. An enzymatic reaction occurring under dimensional restriction conditions (in an intracellular environment) can exhibit noninteger orders. We hypothesize that in a fractal environment, LPS challenge changes the order of the reaction [22]. If this hypothesis is proven, it may partly explain the chemical framework of ET and immunodepression in patients with sepsis.

### Basic considerations leading to the mathematical models

TNF-α production is the result of a chain of enzymatic reactions with a number of intracellular steps. According to Hemker and Hemker [28], this chain of enzymatic reactions behaves like a single enzymatic step [28]. In our model, the chain is resumed in a single reaction in a fractal environment, which led us to the Kopelman fractal-like kinetics model or Savageau power law model with time-dependent exponents.

Also, the actors in an enzymatic reaction are well known to be the substrate, enzyme, product, and substrate-enzyme complex. In our model for the LPS → TNF-α reaction, we identified TNF-α as the product. Furthermore, we considered the endotoxin LPS to be a trigger for the reaction, independent of TNF production, and we modeled it as a given input. Specifically, we considered two possible situations: modeling of LPS as an impulse (approximated by a smooth function) and as a constant function. Elimination of LPS is not directly linked with the production of TNF-α.

The models we considered in this paper may explain ET through the fractal characteristics of the intracellular reaction environment by establishing a link between the reaction environment morphology and intracellular enzymatic reaction rate. ET consists of two successive reactions. This makes the chemical modeling ET especially interesting. Thus, in this approach, the first reaction (specifically, the first LPS challenge) influences the conditions for the second reaction (the second LPS challenge). Both Kopelman’s fractal-like kinetics model and Savageau’s power law model with time-dependent exponents can model this complex behavior.

We used the general Michaelis-Menten mechanism as follows: $S+E\overset{k_{1}}{\underset{k_{-1}}{⇄}}C\overset{k_{2}}{\to }E+P$, in which *S* is the substrate, *E* is the enzyme, *C* is the substrate-enzyme complex, *P* is the product and *k*_1_, *k*_2_, and *k*_−1_ are kinetic constants. We used the same notations for the corresponding concentrations. Moreover, *E*_0_ is the initial enzyme concentration, *E*_*T*_ is the total enzyme concentration, *S*_0_ is the initial substrate concentration and *P*_0_ is the initial product concentration.

### Derivation of the mathematical models

#### Kopelman’s fractal-like kinetics model

**Figure 1:**
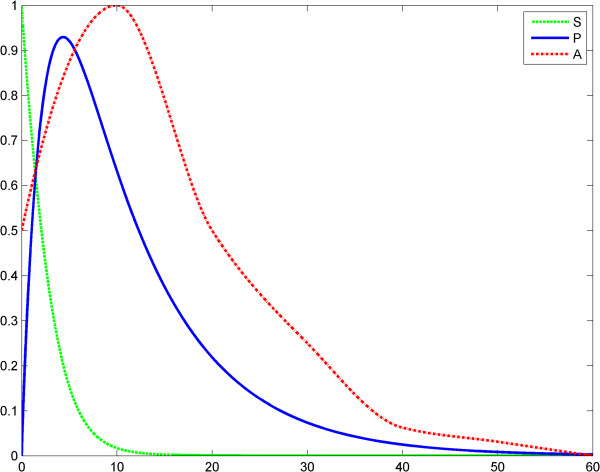
**Fractal like kinetics (model 1), scenario no.1, the LPS is an impulse; first LPS challenge.** Horizontal axes: time (a.u); vertical axes: concentrations (a.u). *A* = LPS, *S* = Substrate, *P* = Product (TNF-α). See also Table 1.

**Figure 2:**
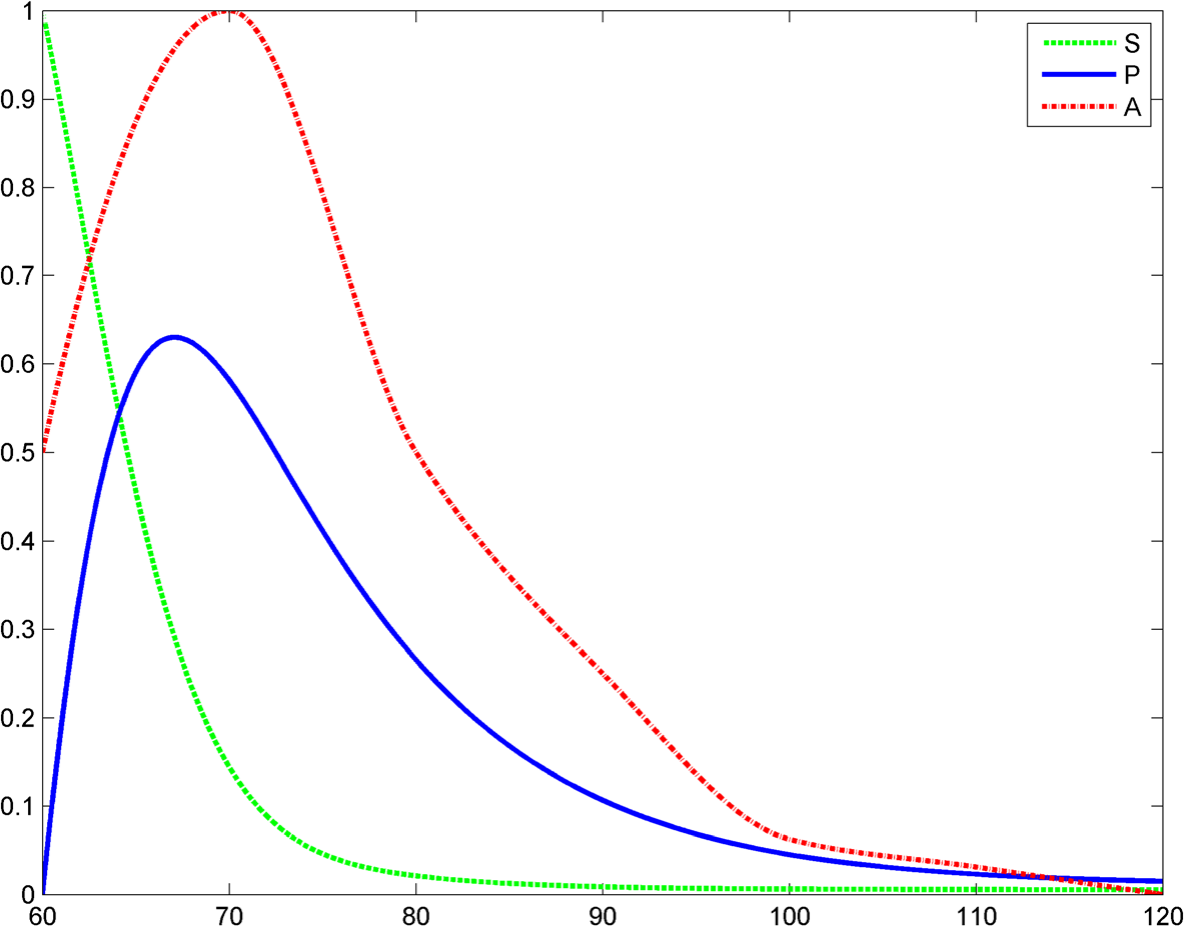
**Fractal like kinetics (model 1), scenario no.1, the LPS is an impulse; second LPS challenge.** Horizontal axes: time (a.u); vertical axes: concentrations (a.u). *A* = LPS, *S* = Substrate, *P* = Product (TNF-α). See also Table 2.

**Figure 3:**
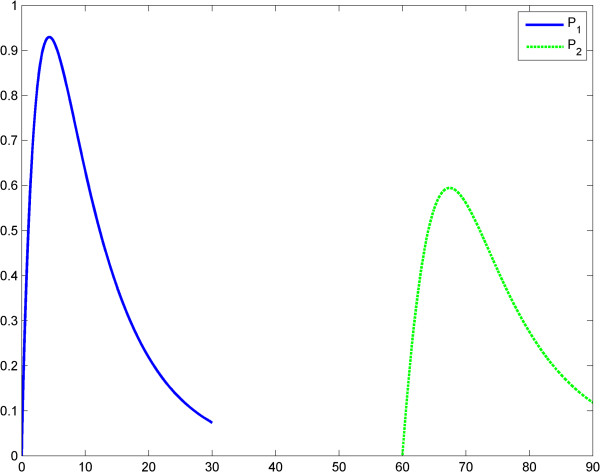
**Fractal like kinetics (model 1); the LPS is an impulse; the first response (*P***_**1**_**) is significantly greater than the second response (*P***_**1**_**).** Horizontal axes: time (a.u); vertical axes: concentrations (a.u). *A* = LPS, *S* = Substrate, *P* = Product (TNF-α).

The above considerations led us to the following (nondimensionalized) ODE model for the TNF-α release under LPS stimulation:

(1) $$\begin{array}{l} \frac{\mathrm{dS}}{\mathrm{dt}}=-A\left(t\right)\;\frac{k_{2}E_{T}S}{S+K_{M}t^{h}}\\ \frac{\mathrm{dP}}{\mathrm{dt}}=\frac{k_{2}E_{T}S}{S+K_{M}t^{h}}-\gamma \;P \end{array}$$

in which *t* is the time, $K_{M}=\frac{k_{-1}+k_{2}}{k_{1}}$ is the Michaelis-Menten constant and *h* is Kopelman's exponent and *γ* is the elimination rate of TNF. Also, in the first equation, the function *A*(*t*) models the LPS evolution. With this model we assessed two scenarios. In scenario 1, the input LPS is an impulse. Specifically, we treated an impulse function as an approximation of the Dirac measure. The first LPS challenge and the evolution of the product (TNF-α) is considered in the interval of 0–60 (arbitrary time units), (Figure 1), whereas the second LPS challenge is considered in the interval 60–120, (Figure 2). The entire evolution of the reaction in the interval 0–120 is depicted in Figure 3. This figure shows and compares the responses of the product (TNF-α) corresponding to two LPS challenges and, accordingly, to ET. The corresponding values of the product are shown in Tables 1 and 2.

**Table 1:** **The values of the Product (TNF**-**α, a.u.) corresponding to the first LPS challenge; model (1), scenario 1**


Time	0	2.38	4.77	7.16	9.55	11.94	14.33	16.72	60
Prod.	0	0.807278	0.925658	0.815613	0.660639	0.519172	0.403355	0.311969	0.003085

**Table 2:** **The values of the Product (TNF**-**α, a.u.) corresponding to the second LPS challenge; model (1), scenario1**


Time	60	62.38	64.77	67.16	69.55	71.94	74.33	76.72	120
Prod	0	0.38829	0.581141	0.630468	0.593811	0.518651	0.434147	0.355385	0.015161

**Figure 4:**
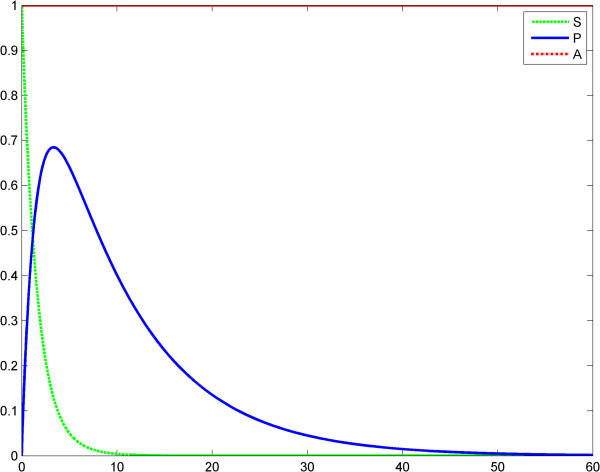
**Fractal like kinetics (model 1), scenario no.2, the LPS is a constant; first LPS challenge.** Horizontal axes: time (a.u); vertical axes: concentrations (a.u). *A* = LPS, *S* = Substrate, *P* = Product (TNF-α). See also Table 3.

**Figure 5:**
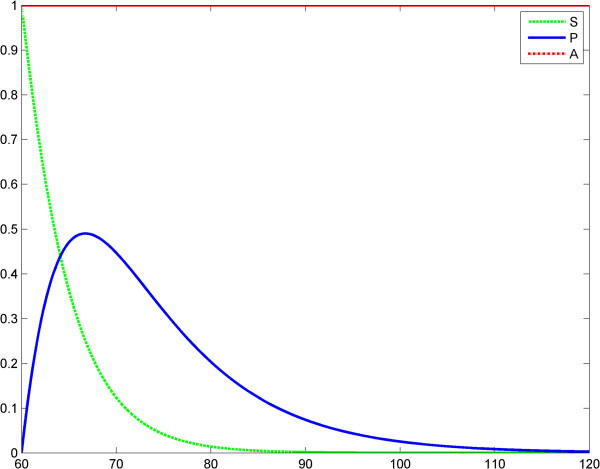
**Fractal like kinetics (model 1), scenario no.2, the LPS is a constant; second LPS challenge.** Horizontal axes: time (a.u); vertical axes: concentrations (a.u). *A* = LPS, *S* = Substrate, *P* = Product (TNF-α). See also Table 4.

**Figure 6:**
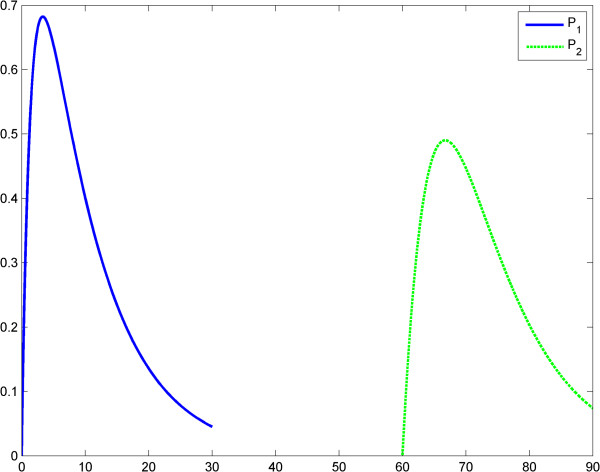
**Fractal like kinetics (model 1), the LPS is a constant; the first response (*P***_**1**_**) is significantly greater than the second response (*P***_**1**_**).** Horizontal axes: time (a.u); vertical axes: concentrations (a.u). *A* = LPS, *S* = Substrate, *P* = Product (TNF-α).

In the second scenario, the input LPS is a time constant. The first LPS challenge and the evolution of the product is considered in the interval 0–60 (Figure 4), whereas the second LPS challenge is considered in the interval 60–120 (Figure 5). The entire behavior of the product in the interval 0–120 is depicted in Figure 6. The values of the product are shown in Tables 3 and 4.

**Table 3:** **The values of the Product (TNF**-**α, a.u.) corresponding to the first LPS challenge; model (1), scenario 2**


Time	0	2.38	4.77	7.16	9.55	11.94	14.33	16.72	60
Prod	0	0.656728	0.650643	0.535076	0.420913	0.326525	0.252014	0.1941	0.001665

**Table 4:** **The values of the Product (TNF**-**α, a.u.) corresponding to the second LPS challenge; model (1), scenario2**


Time	60	62.38	64.77	67.16	69.55	71.94	74.33	76.72	120
Prod	0	0.326552	0.463928	0.489183	0.456542	0.39911	0.335306	0.274702	0.002861

All the simulations were performed using MATLAB (version 7.9.0; MathWorks, Natick, MA). Regarding the coefficient values for all the simulations we performed, the data for the enzymatic reactions were as follows:

*S*_0_ = *S*_*T*_ = 1, *E*_*T*_ = 1, *P*_0_ = 0, *k*_1_ = *k*_− 1_ = 10, *k*_2_ = 1, *K*_*M*_ = 1.1 ([29]). Also, the exponent in the fractal-like kinetics model (1) was *h* = 0.33. The elimination rate for TNF-α was *γ* = 0.11 and the constant function for LPS was *A*(*t*) = 1. Finally, the impulse function for the LPS challenge was a cubic interpolation in MATLAB:

*time* = (*0 10 20 30 40 50 60*), *A* = (*1*/*2 1 1*/*2 1*/*4 1*/*16 1*/*32 0*), *A* = *interp*(*time*,*A*,*t*,‘*cubic*’) and, accordingly, on the time interval 60–120.

### Savageau’s power law model

In Savageau’s power law model below, *t* is the time and is an exponent *g* describing the kinetic order of the enzymatic reaction. Like Savageau, we believe that the kinetic order depends on the fractality of the environment. We therefore considered an enzymatic reaction in a fractal environment using the following equations:

$$\begin{array}{l} \frac{\mathrm{dS}}{\mathrm{dt}}=-k_{1}ES^{g}+k_{-1}C\\ \frac{\mathrm{dC}}{\mathrm{dt}}=k_{1}ES^{g}-\left(k_{-1}+k_{2}\right)C\\ \frac{\mathrm{dP}}{\mathrm{dt}}=k_{2}C\\ E+C=E_{T} \end{array}$$

or:

$$\begin{array}{l} \frac{\mathrm{dS}}{\mathrm{dt}}=-k_{1}\left(E_{T}-C\right)S^{g}+k_{-1}C\\ \frac{\mathrm{dC}}{\mathrm{dt}}=k_{1}\left(E_{T}-C\right)S^{g}-(k_{-1}+k_{2})C\\ \frac{\mathrm{dP}}{\mathrm{dt}}=k_{2}C \end{array}$$

To apply this model to the LPS → TNF-α reaction, we tested two hypothesis: 1) the endotoxin (LPS) acts as a trigger for the reaction, and 2) the product (TNF-α) is eliminated naturally. If *γ* is the rate of the elimination of TNF-α, the system is:

$$\begin{array}{l} \frac{\mathrm{dS}}{\mathrm{dt}}=-k_{1}\left(E_{T}-C\right)S^{g}+k_{-1}C\\ \frac{\mathrm{dC}}{\mathrm{dt}}=k_{1}\left(E_{T}-C\right)S^{g}-(k_{-1}+k_{2})C\\ \frac{\mathrm{dP}}{\mathrm{dt}}=k_{2}C-\gamma \;P \end{array}$$

Our main hypothesis is that at least in the case of an intracellular reaction, the exponent *g* changes over the time. More precisely, *g* = 1 for the first LPS challenge and increases to *g* > 1 before the second LPS challenge. The kinetic order of the reaction is 1 + *g* > 2, [25]. We emphasize that in our hypothesis, *g* increases as a direct consequence of both the first LPS stimulation and the fractal environment. Some coefficients’ time dependence owing to the fractal environment is a feature of Kopelman’s model of fractal-like enzymatic reactions [23]. The new value *g* > 1 remains valid in the second LPS challenge but, if nothing important happens (in the reaction), after a specific interval, the value of *g* will return to 1. With this hypothesis, we suggest the following mathematical model for LPS → TNF-α release:

(2) $$\begin{array}{l} \frac{\mathrm{dS}}{\mathrm{dt}}=-k_{1}\left(E_{T}-C\right)S^{g\left(t\right)}+k_{-1}C\\ \frac{\mathrm{dC}}{\mathrm{dt}}=k_{1}\left(E_{T}-C\right)S^{g\left(t\right)}-(k_{-1}+k_{2})C\\ \frac{\mathrm{dP}}{\mathrm{dt}}=k_{2}C-\gamma \;P \end{array}$$

### The quasi-steady-state-assumption

Adding the quasi-steady-state assumption (QSSA), $\frac{\mathrm{dC}}{\mathrm{dt}}≅0$, to model (2) one gets $C≅\frac{k_{1}E_{T}S^{g}}{k_{1}S^{g}+k_{2}+k_{-1}}$. Just like Kopelman's fractal-like kinetics model (equation 1), we introduce LPS (denoted *A*(*t*)) as a trigger of the reaction. Also, introducing the Michaelis-Menten constant, $K_{M}=\frac{k_{-1}+k_{2}}{k_{1}}$ it results:

(3) $$\begin{array}{l} \frac{\mathrm{dS}}{\mathrm{dt}}=-A\left(t\right)k_{2}\;\frac{E_{T}S^{g\left(t\right)}}{S^{g\left(t\right)}+K_{M}}\\ \frac{\mathrm{dP}}{\mathrm{dt}}=k_{2}\;\frac{E_{T}S^{g\left(t\right)}}{S^{g\left(t\right)}+K_{M}}-\gamma \;P \end{array}$$

The function *A*(*t*) models the LPS evolution during the reaction. In various simulations using MATLAB we found that our model (3) is a good approximation of the model (2). Therefore, the QSSA is a valid hypothesis for the ET phenomenon. We examined the same two scenarios for Savageau model that we did for Kopelman’s model except that we started with the case of a constant LPS, keeping the system autonomous.

**Figure 7:**
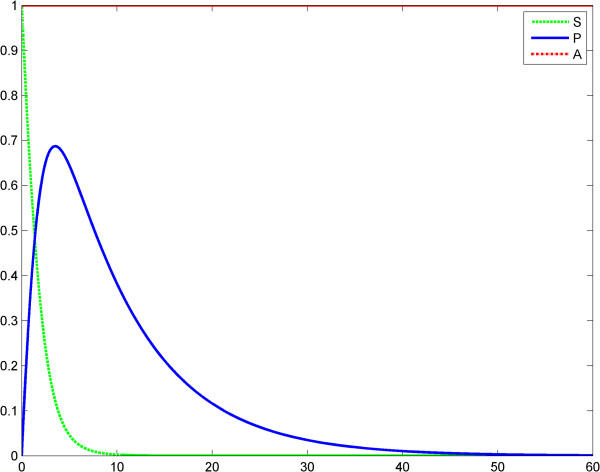
**Power law (model 3), scenario no.1, the LPS is a constant; first LPS challenge.** Horizontal axes: time (a.u); vertical axes: concentrations (a.u). *A* = LPS, *S* = Substrate, *P* = Product (TNF-α). See also Table 5.

**Figure 8:**
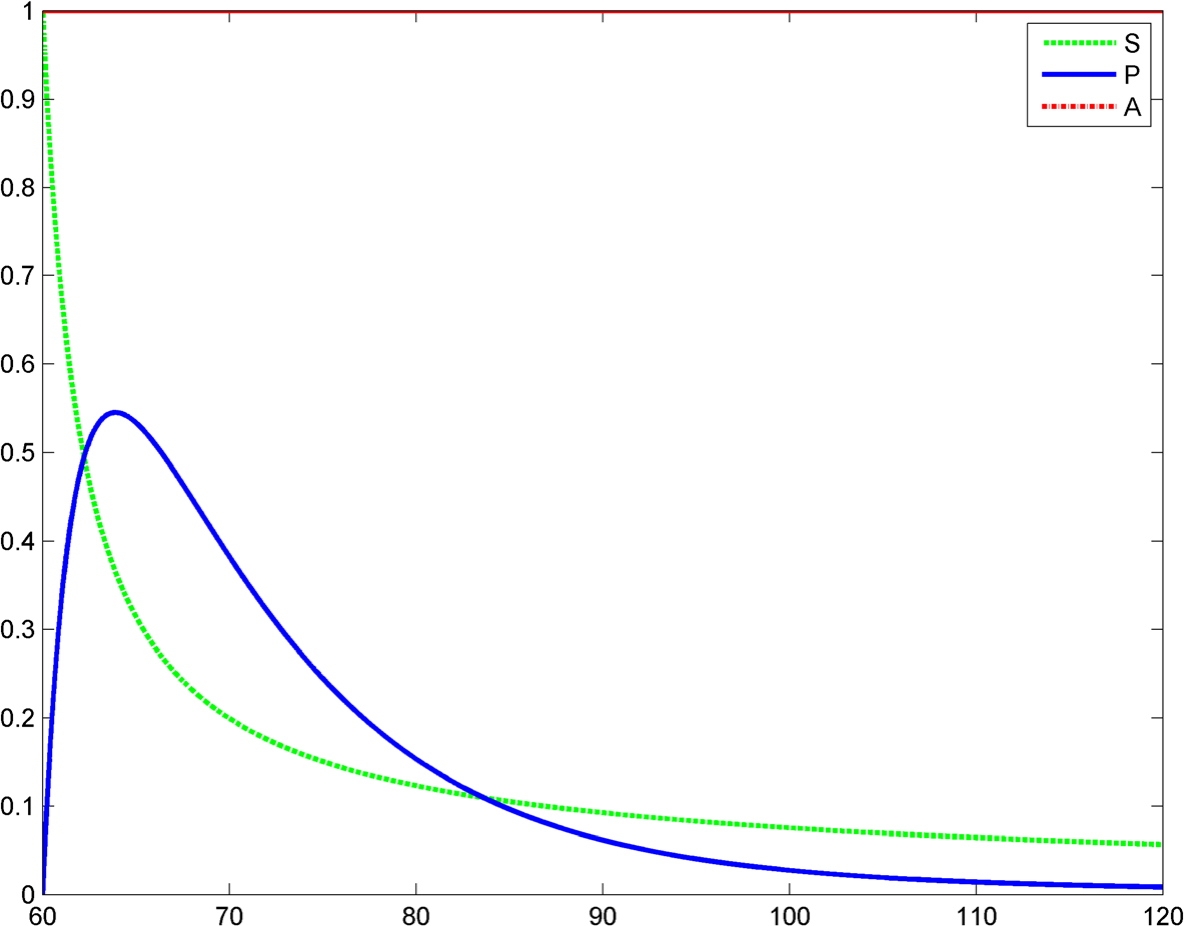
**Power law (model 3), scenario no.1, the LPS is a constant; second LPS challenge.** Horizontal axes: time (a.u); vertical axes: concentrations (a.u). *A* = LPS, *S* = Substrate, *P* = Product (TNF-α). See also Table 6.

**Figure 9:**
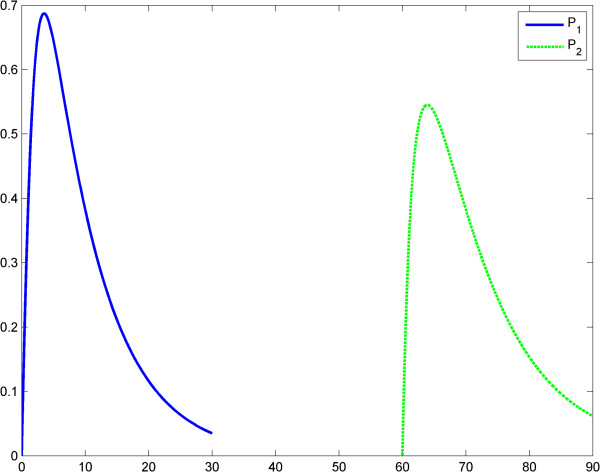
**Power law (model 3), the LPS is a constant; the first response (*P***_**1**_**) is significantly greater than the second response (*P***_**1**_**).** Horizontal axes: time (a.u); vertical axes: concentrations (a.u). *A* = LPS, *S* = Substrate, *P* = Product (TNF-α).

In scenario 1, LPS is constant *A*(*t*) = 1 (Figures 7, 8, 9; Tables 5 and 6). We obtained the following two results using system (3). First, if (*S*, *P*) is a solution of the system (3) with arbitrary positive parameters and the initial conditions *S*(0) = *S*_0_ > 0, *P*(0) = 0; the function *P* has a unique extremum, which is a global maximum. Moreover, the function *S* is strictly decreasing and *lim* _*t*→ *∞*_*S*(*t*) = *lim* _*t*→ *∞*_*P*(*t*) = 0 (the Poincare-Bendixon theorem can be applied [30]). Second, if (*S*_1_, *P*_1_) and (*S*_2_, *P*_2_) are the solutions of system (3) for *g* = 1 and *g* = 2, respectively, with the initial conditions *S*_1_(0) = *S*_2_(0) = *S*_0_ > 0 and *P*_1_(0) = *P*_2_(0) = 0, then

(4) $$P_{1}\left(t\right)-P_{2}\left(t\right)=k_{2}K_{M}E_{T}\;e^{-\gamma \;t}\int_{0}^{t}\frac{\left[S_{1}\left(u\right)-S_{2}^{g}\left(u\right)\right]}{\left[S_{1}\left(u\right),+,K_{M}\right]\left[S_{2}^{g},\left(u\right),+,K_{M}\right]}e^{\gamma \;u}\mathrm{du}$$

and

(5) $$S_{1}-S_{2}^{g}=-\frac{\left(S_{1}+K_{M}\right)\left(S_{2}^{g}+K_{M}\right)}{k_{2}K_{M}E_{T}}\;\left(\frac{dS_{1}}{\mathrm{dt}}-\frac{dS_{2}}{\mathrm{dt}}\right)$$

**Table 5:** **The values of the Product (TNF**-**α, a.u.) corresponding to the first LPS challenge; model (3), scenario1**


Time	0	2.38	4.77	7.16	9.55	11.94	14.33	16.72	60
Prod.	0	0.640502	0.655176	0.526657	0.402921	0.304402	0.229081	0.172155	0.000958

**Table 6:** **The values of the Product (TNF**-**α, a.u.) corresponding to the second LPS challenge; model (3), scenario1**


Time	60	62.38	64.77	67.16	69.55	71.94	74.33	76.72	120
Prod.	0	0.505597	0.537655	0.47516	0.396718	0.32319	0.260281	0.208401	0.008591

*Proof*: Using the second equation of the system (3), the following linear differential equation is obtained by subtraction: $\frac{d\left(P_{1}-P_{2}\right)}{\mathrm{dt}}+\gamma \;\left(P_{1}-P_{2}\right)=k_{2}K_{M}E_{T}\;\frac{S_{1}-S_{2}^{g}}{\left(S_{1}+K_{M}\right)\left(S_{2}^{g}+K_{M}\right)}$. By integrating, one obtains (4). Formula (5) is straight-forward, by using the second equation in (3). We focused on comparing the maximum values of the products *P*_1_ and *P*_2_. Among the possible behaviors of the system (3) we observed two significant ones (depending on *g* and on the initial values of *S*):

1) *P*_1_(*t*) ≥ *P*_2_(*t*), ∀ *t* ≥ 0;

2) ∃ *τ* > 0 such that *P*_2_(*t*) ≥ *P*_1_(*t*), ∀ *t* ≤ *τ* and *P*_1_(*t*) ≥ *P*_2_(*t*), ∀ *t* ≥ *τ*

**Figure 10:**
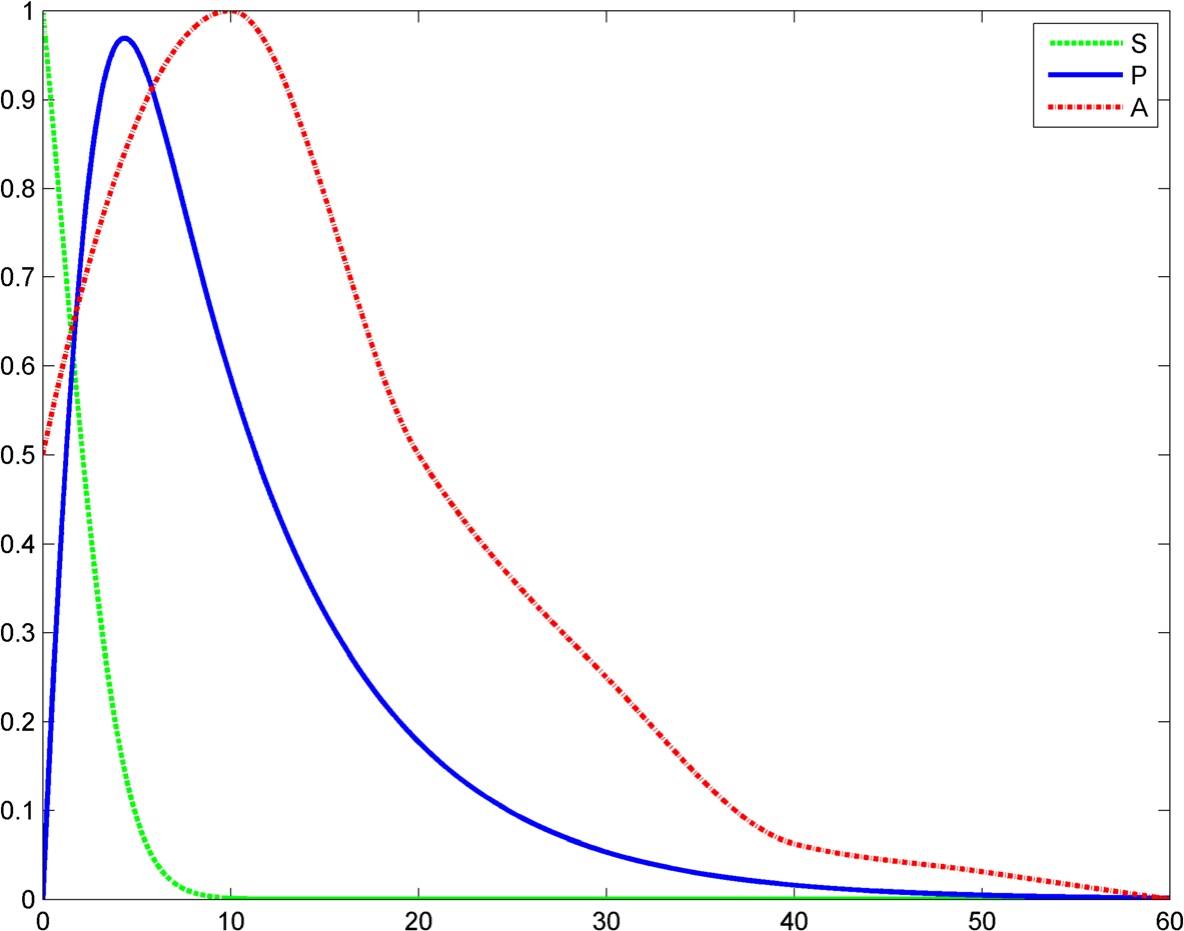
**Power law (model 3), scenario no.2, the LPS is an impulse; first LPS challenge.** Horizontal axes: time (a.u); vertical axes: concentrations (a.u). *A* = LPS, *S* = Substrate, *P* = Product (TNF-α). See also Table 7.

**Figure 11:**
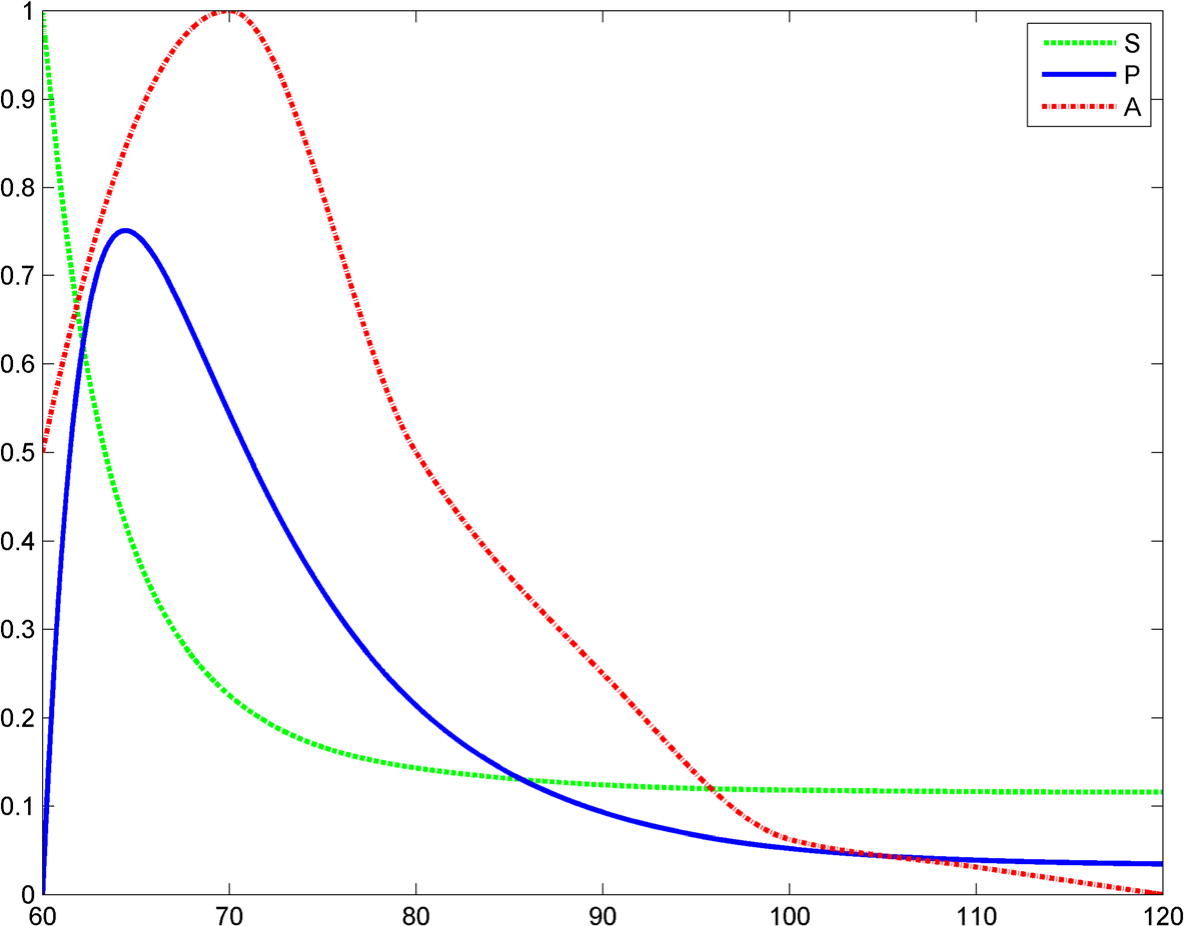
**Power law (model 3), scenario no.2, the LPS is an impulse; first LPS challenge.** Horizontal axes: time (a.u); vertical axes: concentrations (a.u). *A* = LPS, *S* = Substrate, *P* = Product (TNF-α). See also Table 8.

**Figure 12:**
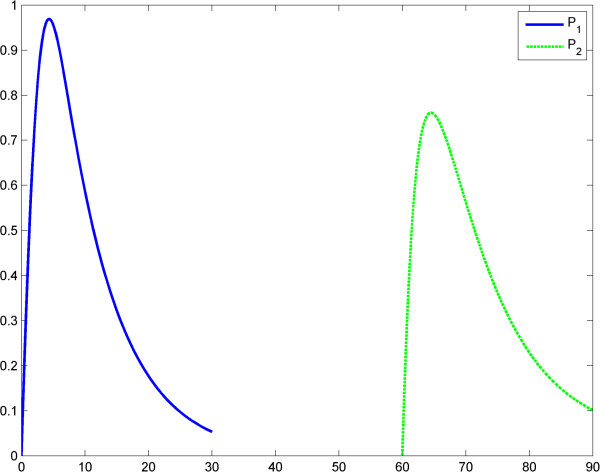
**Power law (model 3), the LPS is an impulse; the first response (*P***_**1**_**) is significantly greater than the second response (*P***_**1**_**).** Horizontal axes: time (a.u); vertical axes: concentrations (a.u). *A* = LPS, *S* = Substrate, *P* = Product (TNF-α).

In the second case, any relation between the maximal values of *P*_1_ and *P*_2_ is possible: *P*_1_^*Max*^ > *P*_2_^*Max*^ or *P*_2_^*Max*^ > *P*_1_^*Max*^. ET is related to the behaviors above (case *P*_1_^*Max*^ > *P*_2_^*Max*^ for the second behavior). This is shown in Figures 7, 8, 9 and Tables 5 and 6. In scenario 2, the input LPS is an impulse (approximated by a smooth function). The first LPS challenge and the evolution of the product (TNF-α) for *g* = 1 is studied in the interval 0–60 (Figure 10), and the second LPS challenge for *g* = 2.5 is studied in the interval 60–120 (Figure 11). The entire behavior in the interval 0–120 is shown in Figure 12. The corresponding values of the product (TNF-α) are listed in Tables 7 and 8, respectively. The exponents in the model (3) are *g* = 1 at the first LPS challenge and *g* ∈ (1.5, 2.5) at the second LPS challenge. All other data in the simulations are the same as in the fractal-like kinetics model above.

**Table 7:** **The values of the Product (TNF**-**α, a.u.) corresponding to the first LPS challenge; model (3), scenario2**


Time	0	2.38	4.77	7.16	9.55	11.94	14.33	16.72	60
Prod.	0	0.791142	0.950024	0.788894	0.597031	0.444241	0.329685	0.244569	0.001095

**Table 8:** **The values of the Product (TNF**-**α, a.u.) corresponding to the second LPS challenge; model (3), scenario2**


Time	60	62.38	64.77	67.16	69.55	71.94	74.33	76.72	120
Prod.	0	0.65047	0.749334	0.675855	0.564764	0.457377	0.365682	0.291171	0.034795

## Discussion

Endotoxin tolerance has been associated with the upregulation of negative regulators like IRAK-M, ST2, SOCS1, and SHIP as well as with the dysregulation of TLR4 [1]. The TLR4 pathway employs signaling through two distinct adaptors, MyD88 and TRIF. The MyD88 pathway leads to the activation of the transcription factor NF-kB and the transcription of inflammatory genes like TNFA, IL1B, IL6 and IL12B. The TRIF pathway triggers activation of the transcription factors IRF3 and STAT1 which, in turn, induce the expression of IFNb and interferon-inducible genes like CCL5 and CXCL10 [1]. A dysregulation of the cytokine releasing capacity of monocyte/macrophages or dendritic cells similar to ET is linked to “immunosuppression” and mortality of sepsis patients. In this so called “immunosuppression” observed in the septic immune response some anti-inflammatory mediators such as IL-10 or TGF beta may also be involved. However, despite recent progress in understanding the molecular basis of ET, there is no consensus yet on the primary mechanism responsible for ET. In this study, we examined the consequences of the fractal-like kinetics of intracellular enzymatic reactions on the LPS signaling machinery.

ET is a particular case of preconditioning [31-33] and has been linked with the down-regulation of the LPS signaling pathway.

LPS signaling (cytokine release under LPS stimulation) can be viewed as a chain of enzymatic reactions. Most of these reactions take place in the intracellular environment. In theory, the kinetics of intracellular enzymatic reactions follows the well-known Michaelis-Menten law. However, researchers formulated most kinetics laws by studying the behavior of simple *in vitro* enzymatic systems in which chemical reactions are considered to take place under diffusive stirring of reactants in a homogenous, dilute environment [10].

Macromolecules obviously exist under completely different conditions *in vitro* and *in vivo*. In the latter, they are located in compartments or on compartment boundaries and are surrounded by a variety of small mobile solutes. This heterogeneity means that the important parameters for chemical reactions *in vivo* are not only the concentrations of reactants and products but also the presence of electric fields, gradients of solute activity (including pH gradients), and transport and reaction rates [34].

The intracellular environment has some particularities that are not seen in *in vitro* biochemical reactions, as macromolecular crowding and hindered diffusion of reactants by compartmentalization (e.g., heterogeneity, inhomogeneity). The finding that cytoskeleton components may undergo fractal organization as percolation clusters gives rise to the interesting possibility that living cells behave according to the principles of fractal geometry [19]. Taking into account the heterogeneity of the intracellular environment, we suggest that fractal-like kinetics explains, at least in part, the chemical framework of ET.

In line with results of previous studies [31-33], we consider ET to be a particular type of preconditioning. To gain further understanding of ET, in the present study, we assessed Kopelman’s fractal like-kinetics and Savageau’s power law models using time-dependent exponents. Furthermore, we added elimination of the product to both models. We took into account the possibility of a changing degree of macromolecular crowding between the first and second LPS stimuli. In other words, we tested the hypothesis (strongly supported by experimental data reported by Hiroi [22]) that between the two LPS challenges, the degree of diffusion limitation increases and the spectral dimension changes; consequently, the power law exponents increase.

The power law exponents are time-dependent as a consequence of a changing degree of macromolecular crowding under different physiological and even pathological circumstances. Hiroi [22] were the first to show that changing the reaction rate may be possible when the degree of intracellular macromolecular crowding is modified by experimentally manipulating the structure of the cytoskeleton. Modifications of the cytoskeleton structure seem to be well established in macrophages in the presence of LPS [35-37]. In addition, Schnell and Turner [10] and Grima and Schnell [38] compared the Kopelman and Savageau models, correlating the fractal-like kinetics of intracellular enzymatic reactions with macromolecular crowding.

In our version of Savageau’s power law model, the time dependence of the exponents is not entirely depicted as a function of time. Future experimental data may bring new light to this matter. However, this is not a limitation of the model, because the events are triggered by LPS challenge (exogenous variable).

### Discussion of the mathematical models of enzyme kinetics

As described above, the aim of this study was to develop a mathematical model able to explain or simulate a component of ET. We proposed that the intracellular fractal environment (macromolecular crowding) contributes to ET and developed two mathematical models of enzyme kinetics: one based on Kopelman’s fractal-like kinetics framework [24] and the other based on Savageau’s power law model of fractal-like kinetics.

The Kopelman model is based on four main ideas. 1) Use the time-depending rate for the Kopelman fractal-like kinetics. 2) Use variable exponents but an autonomous model in Savageau’s power law model. 3) In both cases, after showing that the QSSA hypothesis provides good approximations of the general reaction kinetics systems, use only QSSA models for simulations. 4) ET is mathematically expressed by the difference between the maximum values of TNF-α released in two consecutive stimulations with LPS. Two scenarios are considered: LPS as an impulse function (i.e., an approximation of the Dirac measure) or a constant function.

#### Discussion of Kopelman’s model fractal-like kinetics framework

We think that Kopelman’s model provides a good image of the potential influence of a fractal intracellular environment (macromolecular crowding) on ET. We assume that at the second LPS challenge, although the initial conditions and elimination of the substrate and product are the same as for the first challenge, the first reaction influences the second one, through the values of the rate *k*_1_*t*^− *h*^ (history matters!). Results of simulations show reduced TNF-α release after the second LPS challenge (Figures 3, 4, 5, 6). The value *h* = 0.33 was reported by Hiroi et al. [22].

### Discussion of Savageau’s fractal-like kinetics framework

After the numerical simulations, a more complex (than in Kopelman’s model) behavioral problem with respect to ET was detected. Specifically, we compared the maximum values of TNF-α release. Depending on the coefficients and on the initial conditions the maximum value of the second release of the product was significantly lower than the maximum value of the first release of the product. However, rate value of the second release of the product may be greater than the rate value of the first release of the product for a certain period. To include “history” in Savageau’s model, we chose to change the exponent of the substrate from *g* = 1 at the first LPS challenge to *g* > 1 at the second. This was in line with the data reported by Hiroi et al. [22] supporting a change in the degree of macromolecular crowding after experimental cytoskeleton depolymerization and with observations reported by Voit [39]. This model also partially explains ET. (Figures 9, 10, 11, 12, Tables 5, 6, 7, 8).

### Discussion of the biological relevance of the model

ET is a well studied phenomenon. The role of the negative regulators like IRAK-M, ST2, SOCS1 [1] in the mechanism governing ET has been thoroughly characterized by experimental studies. The mechanistic basis and multi-leveled regulation of endotoxin tolerance in monocyte/macrophages was explained and modeled [8,40-42]. The contribution of these “break” mechanisms is undeniable and it probably represents the main pathway that leads to tolerance.

However, we must consider the fact that these chemical reactions take place in an intracellular environment. This has major implications since it has been shown that macro-molecular crowding (MMC) is the main factor influencing reaction kinetics. Hiroi *et al*. [22] bring convincing arguments in this direction and the idea proposed by Kopelman [23], that fractal-like kinetic (FLK) significantly influences intracellular chemical reactions, is also hard to ignore. MMC in the intracellular environment is a widely accepted reality and it is sustained by experimental evidence that leave little room for doubt [43-45]. Our model is meant to draw attention to the similarities between fractal-like chemical kinetics which this special reaction environment imposes and the negative preconditioning phenomena out of which endotoxin tolerance is best studied. Furthermore, we discuss the influence that the organisation of the intracellular environment has on the cellular response to different challenges. This could eventually lead to the possibility of influencing the immune response by modifying the inner structure of the cell [46,47].

Beyond the details of mathematical formalism, this new point of view underlines the fact that even the passage of time can influence the reaction yield. The “history” of the chemical system under discussion also plays a role and time is, in keeping with Kopelman’s philosophy, the leading actor in this scenario.

It is obvious that time is a crucial factor in endotoxin tolerance, since the cell behaves differently when receiving two identical stimuli separated by a certain time interval. It is here too that “history” matters.

Our FLK-based model does not minimize the role of the numerous negative regulatory factors. It simply draws attention to the fact that macromolecular crowding can contribute significantly to the induction of endotoxin tolerance by imposing geometric constrains and a particular chemical kinetic for the intracellular reactions.

Besides homologous tolerance, LPS priming of the immune cells results in diminished cytokine response after subsequent stimulation with non-LPS stimuli aspeptidoglycan (PGN), lipoteichoic acid (LTA), Pam3CSK4CysSerLys4 (Pam3CSK4), and flagellin, plus such cytokines as TNF-α or IL-1b [48]. Cells treated first with bacterial lipoprotein or MALP-2 (both TLR2 ligands) did not respond to subsequent LPS stimulation and cells pretreated with LPS did not respond to LTA or flagellin [49].

Different ligands can substitute for each other and sometimes mediate cross-tolerance both in vitro and in vivo and no qualitative differences could be observed [50]. This is known as LPS-induced cross-tolerance and has also been observed in association with cells from septic patients. Similar to “classical” endotoxin tolerance models, cross-tolerance has also been explained through mechanistic models based on the multiple negative feedback loops involved in LPS signaling. However, they are less convincing since this phenomenon appears to be less specific. It is understandable that our model may shed a new light on the mechanisms of cross tolerance precisely owing to the fact that it is non-specific. If we accept that macro-molecular crowding can influence the kinetics of intracellular reactions as shown above, then a model based on fractal-like kinetics can contribute to the understanding of very complex phenomena.

It may be stated that FLK acts as a supplementary negative regulator, thus contributing to endotoxin tolerance. In the model we propose it appears as a background chemical kinetic on which the actions of the other factors (which have been “mechanistically” described) are superimposed. It is difficult to ascertain whether FLK determines or simply favors this mechanism.

## Conclusion

The model highlights the links between the organization of the intracellular environment, MMC and the LPS signaling machinery leading to ET. Our FLK-based model does not minimize the role of the numerous negative regulatory factors. It simply draws attention to the fact that macromolecular crowding can contribute significantly to the induction of endotoxin tolerance by imposing geometric constrains and a particular chemical kinetic for the intracellular reactions.

## Competing interests

The authors declare that they have no competing interests.

## Authors’ contributions

CV conceived and designed of the study; final manuscript revision. MO conceived and designed of the study, conceived mathematical model, mathematical simulation; final manuscript revision. PF conceived and designed of the study, conceived mathematical model, mathematical simulation; final manuscript revision. GC conceived and designed of the study; final manuscript revision. All authors read and approved the final manuscript.
